# Breast cancer mortality with varying invitational policies in organised mammography

**DOI:** 10.1038/sj.bjc.6604203

**Published:** 2008-01-29

**Authors:** T Sarkeala, S Heinävaara, A Anttila

**Affiliations:** 1 Finnish Cancer Registry, Helsinki, Finland

**Keywords:** effectiveness, mammography, breast cancer, screening, policy, epidemiology

## Abstract

We examined the effect of different invitational policies on the reduction of breast cancer mortality at 60–79 years of age within the Finnish mammography programme in 1992–2003, which varied in its coverage at 60–69 years of age. The data from 260 municipalities were grouped into three categories: regular invitations at 50–59 years of age only, regular invitations at 50–69 years of age, and regular invitations at 50–59 years of age with irregular invitations at 60–69 years of age. Observed deaths from breast cancer were compared to those expected without screening among all women and among the screened and non-screened women. Observed deaths were obtained from population data and from a cohort follow-up in 1992–2003. Expected deaths were derived by modelling breast cancer mortality at population level in 1974–1985 and 1992–2003. The reduction in breast cancer mortality was strongest, 28% (0.72, 0.51–0.97), in municipalities with regular invitations at 50–69 years of age. No overall effect at 60–79 years of age was observed with regular invitations at 50–59 years of age. The study confirms a reduction by screening of breast cancer mortality in Finland. Uniform extension of invitations to 60–69 years of age would increase the number of prevented breast cancer deaths among the elderly.

Randomised trials of mammography screening have shown that invitation to screening is associated with reduction in breast cancer mortality. An overview of the Swedish randomised trials found a significant 21% reduction among women aged 40–74 years at entry, with the greatest reduction of 33% among those aged 60–69 years at entry (Nyström *et al*, 2002).

After randomised trials, mammography screening has been delivered routinely in several European countries, with emphasis on monitoring the mortality reduction within service screening. Results from Denmark, Sweden, and Finland showed that organised mammography has been effective in reducing breast cancer mortality among screening invitees and participants (Olsen *et al*, 2005; SOSSEG, 2006; Sarkeala *et al*, 2008). The overall reduction in breast cancer mortality in Finland was concentrated at the age group of 50–69 years at death, while in other European countries it was evident at 50–79 years of age.

There has been a marked variation in the invitational age among the municipalities within service screening in Finland: the overall invitational coverage has been over 95% in women aged 50–59 years, but 40% in those aged 60–64 years, and only 20% in those aged 65–69 years (Sarkeala *et al*, 2004). Finland, therefore, provides an opportunity among 60- to 69-year-old invitees for investigating the effect of organised screening independent of other changes in health care.

We examined the consequences of different invitational policies in organised mammography screening on breast cancer mortality among women aged 60 years or more, together with the mortality patterns among screened and non-screened women.

## MATERIALS AND METHODS

The population-based breast cancer-screening programme in Finland was initiated in 1987 with a group-randomised design (Hakama *et al*, 1997). In 1992, all women aged 50–59 years were covered and a Bylaw on Public Health was announced. The law entitled Finnish municipalities to invite women aged 50–59 years to free mammography screening every second year; for those aged 60–69 years, the screening remained optional. The outcome of this decision was a wide variation in the invitational coverage of organised mammography screening at 60–69 years of age.

For the current study, we checked all registered data on biannual invitations to the screening programme in 1992–2003. The only screening providers sending information regularly to the Mass Screening Registry were centres of the Cancer Society of Finland (CSF), covering 260 municipalities, approximately 50% of all activities of the population-based mammography in Finland in 1992–2003.

We divided the data into three invitational categories: regular invitations at the age of 50–59 years (regular 50–59), regular invitations at the age of 50–69 years (regular 50–69), and regular invitations at the age of 50–59 years with irregular invitations at 60–69 years of age (irregular 50–69). The categorisation was based on all available information on invitations in the CSF municipalities in 1992–2003.

These data were available at the individual level. Some CSF municipalities also sent invitations to self-paid mammography for 60- to 69-year-old women in the study period. The data on these biannual invitations with a fee were derived at municipal level from the archives on invited birth cohorts from the Mass Screening Registry.

Most of the CSF municipalities (216) invited 50- to 59-year-old women regularly and 60- to 69-year-old women irregularly to mammography screening in 1992–2003. In these municipalities, only part of women aged 60–69 years received invitations to biannual screening throughout the study period. Eight municipalities invited 50- to 69-year-old women regularly, while 36 restricted their invitations to 50- to 59-year-old women in 1992–2003.

We investigated the impact of different invitational policies on breast cancer mortality among whole female population and among screened and non-screened women in the age group of 60–79 years at death in 1992–2003. The observed breast cancer deaths with screening were compared with the expected breast cancer deaths without screening. The person-years and the observed deaths for the whole female population were derived from population level data. The population data consisted of the number of women and deaths from breast cancers at 60–79 years of age in 1992–2003. The breast cancers had been diagnosed at the age of 50 years or more in 1992–2003. The person-years and the observed deaths for the screened women were obtained from a cohort of screening invitees. The data consisted of person-years and deaths from breast cancer at 60–79 years of age from the individual follow-up. The breast cancers had been diagnosed during the follow-up at the age of 50 years or more. The entry date for the follow-up was 1 January in the first invitation year in 1992–2003. The exit date was the date of death, the date of emigration, or 31 December 2003. The screened women were those who had been invited at least once to any of the screening centres of the CSF and who had participated after their first invitation in the study period. Detailed description on the formulation of cohort was given in a previous study (Sarkeala *et al*, 2008).

The person-years and the observed deaths for the non-screened women were obtained by subtracting the figures of the screened women from those of the whole female population. The group ‘non-screened’ thus contained the non-participants and the non-invitees of organised mammography screening within the CSF centres in 1992–2003.

The expected deaths without screening were derived by multiplying the expected breast cancer mortality rates without screening with the corresponding person-years at risk. As non-invited controls were not available, the expected breast cancer mortality rates without screening were estimated by modelling, using population data from 1974 to 1985 and from 1992 to 2003. The population data consisted of the number of women and deaths from breast cancers at 60–79 years of age in 1974–1985 and 1992–2003. The breast cancers had been diagnosed at the age of 50 years or more in 1974–1985 and in 1992–2003.

The population data were derived from the Finnish Cancer Registry and the National Population Registry. The individual data were collected from the Finnish Mass Screening Registry and were linked with the data from the Finnish Cancer Registry and the National Population Registry using a personal identifier as a key.

The population data from 1975 to 1985 and from 1992 to 2003 were used to estimate the expected mortality rates without screening in 1992–2003. The period 1974–1985 represented the latest possible prescreening era of equal length to the screening period 1992–2003: some CSF municipalities had started screening 1 year before the launch of the national programme.

Expected breast cancer mortality rates among the whole female population and among the screened and non-screened women were modelled by Poisson regression with a logarithmic link function. Five-year age groups at death (60–64, 65–69, 70–74, 75–79), policy categories (regular 50–59, regular 50–69, irregular 50–69), and calendar time within the two periods (1, 2, …, 12 years) were used as explanatory variables in both models. For the entire female population, the period before (1974–1985) and with screening (1992–2003) by 10-year age groups at death was included in the model. In the model for screened and non-screened women, the period before (1974–1985) and with screening (1992–2003) by 5-year age groups at death and screening indicator (screened, not screened) were included. Models are described in detail in Appendix.

The average, category-specific difference in breast cancer mortality between the two 12-year periods (1974–1985 and 1992–2003) among the whole female population (model 1), and between the screened and non-screened women (model 2), represented the screening effect. The fitted mortality values of the model were calculated after excluding the screening effect, representing the expected breast cancer mortality rates without screening. Further details are presented in Appendix.

The confidence intervals of the effect estimates (observed with screening in 1992–2003/expected without screening in 1992–2003) were corrected with overdispersion constants produced by the two models (1.39/1.25). The bias due to self-selection among screened women was adjusted by a described by Cuzick and co-workers method (Cuzick *et al*, 1997).

## RESULTS

In 1992–2003, there were altogether 2 388 775 person-years in the age group 60–79 (Table 1). The overall reduction in breast cancer mortality in the age group of 60–79 years at death was 15% (relative risk 0.85, 95% confidence interval 0.77–0.93). In municipalities that had invited 50- to 69-year-old women regularly, the breast cancer mortality reduced by 28% (0.72, 0.51–0.97) (Table 2). The strongest reduction (32%) was estimated in the age group of 70–79 years at death. In municipalities that had invited women aged 50–59 years only, no overall reduction in breast cancer mortality at 60–79 years of age could be observed.

Almost 80% of person-years in the study were accumulated from municipalities that had invited 50- to 59-year-old women regularly and 60- to 69-year-old women irregularly to mammography screening (Table 1). In this invitational category, the overall reduction in breast cancer mortality in the age group of 60–79 years at death was 16% (0.84, 0.75–0.92) (Table 2). The reduction was strongest (24%) in the age group of 60–69 years at death. The overall effect estimates of municipalities inviting 50- to 69-year-old women regularly or irregularly differed significantly from those of the invitational category ‘regular 50–59’ (*P*=0.003).

In the cohort of screened women, there were altogether 1 023 598 person-years during the follow-up (Table 1). A reduction in breast cancer mortality was observed in all invitational categories (Table 3). In municipalities that invited women aged 50–59 years regularly, the reduction could be observed in the age group of 60–69 years at death. In municipalities that invited women aged 50–69 years, the reduction was observed in the age group of 60–79 at death. The non-adjusted relative risk was 0.62 (0.34–0.99) and adjusted relative risk was 0.67 (0.34–0.99). Owing to widespread non-registered screening with a customer fee in municipalities offering irregular screening at 60–69 years of age, the overall relative risk among the non-screened was slightly below 1, leading to overestimations in adjusted efficacy estimates of the women in this category.

## DISCUSSION

We investigated the impact of organised mammography on breast cancer mortality by comparing the observed deaths from breast cancer with screening to those expected without screening within three invitational policies in the age group of 60–79 years at death in 1992–2003. The overall reduction in breast cancer mortality was strongest, 28%, in municipalities that had invited 50- to 69-year-old women regularly to mammography screening. With regular invitations at the age of 50–59 years only, no reduction was observed in the age of 60–79 years at death. Among the screened women, the overall reduction in breast cancer mortality was at similar levels in all three invitational categories, but the effect became evident at different ages.

The data for the effect estimates were derived from the Finnish Cancer, the National Population, and the Mass Screening Registries, which covered all the municipalities under study, and provided reliable data on breast cancer diagnoses and deaths from breast cancer, as well as complete information on invitations and visits to the organised programme.

The invitational coverage of mammography screening with a customer fee was approximately 25% among 60- to 69-year-old Finnish women in the late 1990s (Saarenmaa *et al*, 2000). The proportion of attendees (40–70%) was lower than that in the organised programme (88–93%) (tabulated data from the Mass Screening Registry; Sarkeala *et al*, 2004). Higher socioeconomic status did not affect attendance (Immonen-Räihä *et al*, 2001). Individual level information on invitations to mammography screening associated with a fee was not available in the Mass Screening Registry. This hampered the investigation of screening effect, especially in the invitational category ‘irregular 50–69’, and led to underestimations of the relative risks among the non-screened women in this policy category. Non-invitational (opportunistic or diagnostic) mammography, which has so far not been registered in Finland, could also modify the effect estimates. This activity was most prevalent at 60–69 years of age in urban communities without organised screening.

Evidence-based guidelines for current care have been distributed by the Finnish Medical Society to all professionals via a health portal (http://www.terveysportti.fi) since 1994, and no systematic variations in treatment or diagnostic services between the Finnish municipalities have been reported. The unique organisation of mammography in Finland thus enabled us to investigate the impact of screening on breast cancer mortality as a natural experiment, without other activities in health care influencing the effect estimates. The overall results, with a clear variation in breast cancer mortality between the different invitational policies, confirm the previously addressed impact of organised screening breast cancer mortality among screening invitees and participants in Finland (Sarkeala *et al*, 2008).

The invitational coverage has varied widely among municipalities, Finland. In other European countries, mammography programmes have covered women aged 50–69 years. In Sweden, where women aged 40–69 years have been invited regularly to biannual screening from the beginning of the programme, the overall reduction in breast cancer mortality among screening invitees has been higher than in Finland, 27% (SOSSEG, 2006). In Denmark, with invitations to 50- to 69-year-old women, the mortality reduction among invitees in the Copenhagen area was 25% at 50–79 years of age being strongest, 42 and 31% at 70–74 and 75–79 years of age, respectively (Olsen *et al*, 2005).

The considerable post-screening follow-up has reduced the estimated overall screening effect in the age group of 60–69 years at death in Finland (Sarkeala *et al*, 2008). In the municipalities that invited women aged 50–69 years regularly to screening, the estimates were, however, consistent with those from Denmark at the age group of 60–79 years at death and from Turku, Finland (Olsen *et al*, 2005; Parvinen *et al*, 2006). The results were also consistent with previous predictions on breast cancer mortality at 50–69 years of age (Hristova and Hakama, 1997).

Few municipalities invited only 50- to 59-year-old women to biannual mammography screening in the study period 1992–2003. Instead, most had invited women aged 50–59 years regularly and those aged 60–69 years irregularly. Nevertheless, with regular invitations of women aged 50–69 years in the study period, the additional number of breast cancer deaths prevented at 60–79 years of age would have been approximately 140 in the municipalities under study. Uniform extension of invitations would thus almost have doubled the number of prevented breast cancer deaths at 60–79 years of age (see Table 2).

Since 1992, the age limits of organised breast cancer screening have been subject to a continuous debate in Finland. In December 2006, the Ministry of Health Care and Social Welfare of Finland announced a Bylaw on Screening to extend the invitational age from 50–59 to 50–69 years, the expansion to be done gradually: in 2007, the women born in 1947 will be invited. Thereafter, all younger birth cohorts will receive a personal invitation to free-of-charge mammography screening every second year until 69 years of age. Our findings suggest that this gradual expansion of invitations will increase the overall number of prevented breast cancer deaths at 60–79 years of age, but that the annual increase will remain rather small. The cost-effectiveness of this expansion is difficult to measure because no baseline estimates on the cost-effectiveness of opportunistic screening in women in the age group of 60–69 years exist.

Our results confirm the impact of organised mammography screening on breast cancer mortality in Finland. Uniform extension of invitations to women aged 60–69 years, as recommended on the basis of randomised trials, will increase the number of deaths prevented at 60–79 years of age.

## Figures and Tables

**Table 1:** Number of municipalities and person-years at risk among all female population, and among screened and non-screened women in each policy category in 1992–2003

		**Person-years**		
**Policy category**	**Number of municipalities**	**All women**	**Screened**	**Non-screened**
Regular 50–59	36	256 548	86 769.2	169 778.8
Regular 50–69	8	228 527	195 139.3	33 387.7
Irregular 50–69	216	1 903 700	741 689.2	1 162 010.8
All	260	2 388 775	1 023 597.7	1 365 177.3

**Table 2:** Observed and expected numbers of breast cancer deaths, and effect estimates with 95% CIs among all women by policy categories in 1992–2003

	**Regular 50–59**	**Regular 50–69**	**Irregular 50–69**
*60–69 at death*			
Observed	53	35	282
Expected	52.6	45.8	371.6
Observed/expected (95% CI)	1.01 (0.66–1.44)	0.76 (0.45–1.19)	0.76 (0.64–0.89)
			
*70–79 at death*			
Observed	78	40	446
Expected	73.2	58.8	499.3
Observed/expected (95% CI)	1.07 (0.76–1.44)	0.68 (0.42–1.03)	0.89 (0.78–1.01)
			
*60–79 at death*			
Observed	131	75	728
Expected	125.8	104.5	871
Observed/expected (95% CI)	**1.04** (**0.81–1.31)**	**0.72** (**0.51–0.97)**	**0.84** (**0.75–0.92)**

CI, confidence interval.

**Table 3:** Observed and expected numbers of breast cancer deaths, and effect estimates with 95% CIs among screened and non-screened women by policy categories in 1992–2003

	**Regular 50–59**	**Regular 50–69**	**Irregular 50–69**
*Screened*			
60–69 years at death			
Observed	30	28	181
Expected	46.0	36.7	269.9
Observed/expected (95% CI)	0.65 (0.39–1.00)	0.76 (0.45–1.19)	0.67 (0.55–0.80)
			
70–79 years at death			
Observed	0	29	29
Expected	0.0	55.7	61.3
Observed/expected (95% CI)	0.00	0.52 (0.31–0.81)	0.47 (0.28–0.73)
			
60–79 years at death			
Observed	30	57	210
Expected	46.0	92.3	331.2
Observed/expected (95% CI)	**0.65** (**0.39–1.00)**	**0.62** (**0.43–0.85)**	**0.63** (**0.53–0.75)**
			
*Non-screened*			
60–69 years at death			
Observed	23	8	101
Expected	15.6	4.8	98.5
Observed/expected (95% CI)	1.48 (0.82–2.40)	1.67 (0.54–3.73)	1.03 (0.79–1.30)
			
70–79 years at death			
Observed	78	13	417
Expected	63.3	8.6	440.0
Observed/expected (95% CI)	1.23 (0.91–1.61)	1.51 (0.66–2.86)	0.95 (0.84–1.07)
			
60–79 years at death			
Observed	101	21	518
Expected	78.9	13.4	538.5
Observed/expected (95% CI)	**1.28** (**0.99–1.62)**	**1.57** (**0.84–2.61)**	**0.96** (**0.86–1.07)**

CI, confidence interval.

## Appendix

The following definitions were made for the models. Let *i*=1, 2, 3, 4 denote the categorical variable 5-year age group at death (60–64, 65–69, 70–74, 75–79), *P*=1, 2, 3 denote the categorical variable screening policy (regular at 50–59 years of age, regular at 50–69 years of age, irregular at 50–69 years of age), *y* denote the numerical variable calendar year of death, and *n*=1, 2, …, 12 denote the numerical variable calendar time in years since the beginning of the two periods (1974–1985 and 1992–2003). In the period 1974–1985, the variable *n*=*y*−1973, and in the period 1992–2003, *n*=*y*−1991. Thus, *n* is a function of y, that is, *n*=*n*(*y*).

Let us define further the categorical screening effect variable *k*=1, 2, 3 for the whole female population for model 1: *k*=1 in the calendar period 1974–1985, *k*=2 in the calendar period 1992–2003 for the age group 60–69 (*i*=1, 2), and *k*=3 in the calendar period 1992–2003 for the age group 70–79 (*i*=3, 4). Let us also define the categorical screening effect variable *s*=1, …, 9 for the screened and non-screened women for model 2: *s*=1 in the calendar period 1974–1985, *s*=2, 3, 4, 5 for *i*=1, 2, 3, 4 in the calendar period 1992–2003 for the non-screened women, and *s*=6, 7, 8, 9 for *i*=1, 2, 3, 4 in the calendar period 1992–2003 for the screened women.

Among the entire female population, the model for the incidence-based mortality *m*_*ip*y_ in the age group *i*, in the policy category *p*, and in the calendar year *y* can then be written as log(*m*_*ipy*_)=*α*+*β*_*i*_*i*+*γn*(*y*)+*δ*_*i*_*in*(*y*)+*ε*_*p*_*p*+*ζ*_*k*_*k*+*η*_*pk*_*pk* (model 1). Among the screened and non-screened women, the model for the incidence-based mortality is expressed as log(*m*_*ipys*_)=*α*+*β*_*i*_*i*+*γn*(*y*)+*δ*_*i*_*in*(*y*)+*ε*_*p*_*p*+*ζ*_*s*_*s*+*ε*_*p*_*p*+*ζ*_*s*_*s*+*η*_*ps*_*ps* (model 2).

The interactions *in*(*y*), *pk*, and *ps* were included in the two models due to their statistical significance (*in*(*y*)) or due to the question of interest (*pk* or *ps*).

Expected breast cancer mortality rates without screening for the calendar period 1992–2003 were estimated by excluding the variables related to the screening effects, that is, by using exp(*α*+*β*_*i*_*i*+*γn*(*y*)+*δ*_*i*_*in*(*y*)+*ε*_*p*_*p*) with the estimates from the respective models.
